# Burnout, psychological wellbeing, and musculoskeletal complaints in UK GPs: an observational study

**DOI:** 10.3399/BJGPO.2023.0007

**Published:** 2023-11-29

**Authors:** Gregory JH Biddle, Nicholas Thomas, Charlotte L Edwardson, Stacy A Clemes, Amanda J Daley

**Affiliations:** 1 The Centre for Lifestyle Medicine and Behaviour, School of Sport, Exercise and Health Science, Loughborough University, Loughborough, UK; 2 Diabetes Research Centre, University of Leicester, Leicester General Hospital, Leicester, UK; 3 NIHR Leicester Biomedical Research Centre, Leicester, UK; 4 Research, Royal College of General Practitioners, London, UK; 5 Sedentary Behaviour and Health, Diabetes Research Centre, University of Leicester, Leicester General Hospital, Leicester, UK; 6 Sedentary Behaviour and Health, NIHR Leicester Biomedical Research Centre, Leicester, UK; 7 School of Sport, Exercise and Health Science, Loughborough University, Loughborough, UK

**Keywords:** burnout, psychological, exercise, general practice, musculoskeletal health, physical activity, wellbeing

## Abstract

**Background:**

Healthcare systems are under unprecedented pressure. GPs are crucial to the health of the population, yet their own health and wellbeing is often overlooked.

**Aim:**

To investigate feelings of burnout, psychological wellbeing, and musculoskeletal complaints in GPs across the UK and to examine whether these health outcomes vary according to the time GPs spent sitting, their participation in physical activity each day, and the time spent working per day or week.

**Design & setting:**

Observational study involving GPs located across the UK.

**Method:**

An online survey was emailed to working members of the Royal College of General Practitioners and shared on social media between October and December 2020. The survey included questions on burnout, psychological wellbeing, musculoskeletal complaints, sitting time, physical activity, and time spent working. Mean differences were examined for burnout, psychological wellbeing, and musculoskeletal complaints.

**Results:**

Data from 406 GPs showed a high level of burnout (35.5%) and musculoskeletal complaints (neck, shoulder and back: 81.8%; arms: 28.3%; and legs: 49.8%). Psychological wellbeing was low in 22.9% of GPs. Burnout was lower in GPs who met current physical activity guidelines, while psychological wellbeing was higher in those with >2 breaks in sitting per hour. Musculoskeletal complaints were higher in those spending >50% of sitting time in prolonged bouts (≥30 minutes).

**Conclusion:**

A high proportion of GPs reported experiencing burnout and musculoskeletal complaints, but these health concerns were less evident in GPs who spent less time in prolonged sitting, took more breaks in sitting, and who were more physically active.

## How this fits in

Healthcare systems globally are under strain, and poor staff retention, declining wellbeing, and growing pressure of general practice are among the key drivers. In the UK, it has been reported that more than half of GPs plan on leaving the profession in the next 5 years. This study showed high levels of burnout, poor psychological wellbeing, and musculoskeletal complaints among GPs, yet meeting physical activity guidelines and spending less time in prolonged sitting were associated with better health and wellbeing. Workplace initiatives could therefore target these behaviours to improve wellbeing.

## Introduction

Healthcare systems around the world are under immense strain. In the UK, the NHS is facing the most severe pressure in its history as a result of a plethora of long-term issues, including chronic understaffing, poor staff retention, declining wellbeing, and growing pressure on general practice.^1^ Now more than ever, it is important to examine ways in which these issues can be addressed, ultimately leading to improved care for patients and working conditions for individuals working in health care.

GPs are crucial to the health and wellbeing of the population, yet their own health and wellbeing are often overlooked. GPs have more contact with patients than any other group of doctors, with >300 million consultations with the public per year and rising.^2,3^ This workload not only demonstrates the key role GPs play in the health infrastructure, but also points to the substantial psychological demand of the job. Patients are often worried or stressed about their medical needs, which often requires a great deal of psychological energy from the GP to address these concerns. As a result, burnout and psychological ill-health is a considerable risk for GPs. Not only is this a concern for individual GPs, but the potential impact on the whole workforce is profound. A recent study reported that more than half of GPs were planning on leaving the profession within 5 years.^4^ Given current figures, this would mean the UK is due to lose ~25 000 GPs over the next 5 years. Replacing this number will be difficult given the demands placed on individuals in these roles, as well as very costly.

Workload pressures increased across all healthcare settings during the COVID-19 pandemic, including general practice. Given general practice was already working beyond its capacity, this crisis has intensified the issues. Studies have reported that a large number of GPs have experienced burnout and depression during COVID-19,^5,6^ which may deteriorate further once the effects of COVID-19 on primary care services are fully realised over time. Furthermore, changes to the working practices of GPs, such as increased use of remote consultations, which were hastened during the COVID-19 pandemic, may negatively impact the physical activity and sedentary behaviour of GPs, further exacerbating health and wellbeing issues.^7^

This study aimed to assess feelings of burnout, psychological wellbeing, and musculoskeletal complaints in GPs across the UK, and to examine whether these health outcomes vary according to the time GPs spent sitting, their participation in physical activity each day, and the time spent working per day or week.

## Method

### Design and participants

This observational study was conducted as part of a larger programme of work focused on GPs’ health and wellbeing. Some data have been reported previously.^8^ To be eligible to participate in this study GPs had to be working in the UK at the time. The Royal College of General Practitioners (RCGP) sent the survey link by email to members who had previously agreed to be contacted about research. Some GPs were recruited via social media. Data were collected between the 22 October and 11 December 2020. Retired GPs and those not working or fully qualified (that is, medical students) were excluded. All participants provided informed consent.

### Questionnaire development

Data were collected using the Qualtrics online platform. Burnout, psychological wellbeing, and the presence of musculoskeletal complaints were measured using validated questionnaires.^9–11^ Burnout was assessed using a single-item measure, which asked participants to state which statement relating to degree of burnout was most relevant to them. Psychological wellbeing was assessed using the short version of the Warwick–Edinburgh Mental Wellbeing Scale (SWEMWBS), a seven-item questionnaire based in the Rasch measurement model. Musculoskeletal complaints were assessed using the Standardised Nordic questionnaires for the analysis of musculoskeletal symptoms, which asked about presence of pain, discomfort, or numbness in nine areas of the body (neck, shoulder, upper back, elbow, wrist or hand, lower back, hip or thigh, knee, and ankle or feet). If present, the pain is ranked on a scale of 1–10. For this study, these nine areas were grouped to form three larger body groups (1: neck, shoulder, and back; 2: arms; and 3: legs). Physical activity was assessed using the Exercise Vital Sign questionnaire,^12^ which consists of three questions asking the participant the following: 1) on average how many days per week they engage in moderate-to-vigorous intensity physical activity; 2) how many minutes on average at this level is spent per day; and 3) how many days per week do they perform muscle strengthening activity. Sitting variables were assessed using self-reported measures of percentage of occupational sitting time that is prolonged (≥30 minutes) and the number of breaks in sitting per hour during work.^13^ Self-reported data were also collected for demographics (age, gender, and ethnic group) and work-related variables (working country, working location, job role, sessions worked per week, and practice list size).

### Statistical analysis

Data were analysed using IBM SPSS Statistics (version 27). Data are first presented as frequencies to describe participants’ feelings of burnout, psychological wellbeing, musculoskeletal complaints, physical activity, sitting time, working hours, and working days. Independent *t*-tests were used to assess the difference between defined physical activity, sitting and working groups, and burnout and psychological wellbeing scores. χ^2^ tests were used to assess the difference between physical activity, sitting and working groups, and musculoskeletal complaints. Multiple linear regression modelling was also used in a sensitivity analysis to identify predictors (that is, age, gender, ethnic group, and body mass index) of the outcomes of interest (burnout, psychological wellbeing, and musculoskeletal complaints) while keeping other variables constant between work-related variables (that is, working location, job role, qualification year, clinical sessions of work per week, and number of patients registered at the practice). All variables were entered simultaneously into the regression model. Statistical significance was set at α = 0.05.

## Results

### Participant characteristics

For the larger programme of work 14 142 GPs were invited to participate and 810 responded, of whom 445 agreed to answer additional questions about their health and wellbeing, including items relating to burnout, psychological wellbeing, and musculoskeletal complaints. Valid study data were contained in 406/445 responses. Most responders were female (71.4%), from a White ethnic group (80.8%), aged 41–50 years (39.7%), members of RCGP (93.8%), and worked 4–6 sessions per week (58.1%). Most GPs reported being within the healthy body mass index range (57.4%) and 42.6% were classified as overweight or obese. Responses from GPs were recorded from all regions or countries within the UK, with a reasonable split between GPs who worked in cities, towns, and rural settings (Table 1).

**Table 1.** Sociodemographics and work-related variables according to sitting and physical activity status

		Physical activity status, *n* (%)		Prolonged sitting, *n* (%)		Breaks in sitting, *n* (%)	
**Characteristic**	**Total sample** **(** ***N*** **= 406),** ***n*** **(%)**	Meeting guidelines (*n* = 123)	Not meeting guidelines (*n* = 283)	<50% of sitting (*n* = 145)	≥50% of sitting (*n* = 261)	≥2 per hour (*n* = 187)	≤1 per hour (*n* = 219)
**Age, years**							
24–40	96 (23.6)	40 (32.5)	56 (19.8)	24 (16.6)	72 (27.6)	35 (18.7)	61 (27.9)
41–50	161 (39.7)	53 (43.1)	108 (38.2)	55 (37.9)	106 (40.6)	70 (37.4)	91 (41.6)
51–70	149 (36.7)	30 (24.4)	119 (42.0)	66 (45.5)	83 (31.8)	82 (43.9)	67 (30.6)
**Female**	290 (71.4)	86 (69.9)	204 (72.1)	94 (64.8)	196 (75.1)	121 (64.7)	169 (77.2)
**White ethnicity**	328 (80.8)	93 (75.6)	235 (83.0)	125 (86.2)	203 (77.8)	157 (84.0)	171 (78.1)
**Working country**							
England	330 (81.3)	99 (80.5)	231 (81.6)	117 (80.7)	213 (81.6)	150 (80.2)	180 (82.2)
Wales	22 (5.4)	6 (4.9)	16 (5.7)	9 (6.2)	13 (5.0)	12 (6.4)	10 (4.6)
Scotland	45 (11.1)	14 (11.4)	31 (11.0)	18 (12.4)	27 (10.3)	22 (11.8)	23 (10.5)
Northern Ireland	9 (2.2)	4 (3.3)	5 (1.8)	1 (0.7)	8 (3.1)	3 (1.6)	6 (2.7)
**GP working location**							
City	135 (33.3)	44 (35.8)	91 (32.2)	41 (28.3)	94 (36.0)	59 (31.6)	76 (34.7)
Town	206 (50.7)	60 (48.8)	146 (51.6)	76 (52.4)	130 (49.8)	90 (48.1)	116 (53.0)
Rural	65 (16.0)	19 (15.4)	46 (16.3)	28 (19.3)	37 (14.2)	38 (20.3)	27 (12.3)
**Job role**							
Managing/executive partner	19 (4.7)	3 (2.4)	16 (5.7)	9 (6.2)	10 (3.8)	14 (7.5)	5 (2.3)
GP partner	221 (54.4)	68 (55.3)	153 (54.1)	93 (64.1)	128 (49.0)	104 (55.6)	117 (53.4)
Salaried/Locum GP	148 (36.5)	41 (33.3)	107 (37.8)	40 (27.6)	108 (41.4)	62 (33.2)	86 (39.3)
GP specialist	18 (4.4)	11 (8.9)	7 (2.5)	3 (2.1)	15 (5.7)	7 (3.7)	11 (5.0)
**Sessions of work per week**							
1–3	40 (9.9)	12 (9.8)	28 (9.9)	10 (6.9)	30 (11.5)	18 (9.6)	22 (10.0)
4–6	236 (58.1)	62 (50.4)	174 (61.5)	89 (61.4)	147 (56.3)	109 (58.3)	127 (58.0)
≥7	130 (32.0)	49 (39.8)	81 (28.6)	46 (31.7)	84 (32.2)	60 (32.1)	70 (32.0)
**Practice list size**							
<6,000	65 (16.0)	19 (15.4)	46 (16.3)	21 (14.5)	44 (16.9)	36 (19.3)	29 (13.2)
6000–14,999	231 (56.9)	71 (57.7)	160 (56.5)	80 (55.2)	151 (57.9)	104 (55.6)	127 (58.0)
≥15,000	110 (27.1)	33 (26.8)	77 (27.2)	44 (30.3)	66 (25.3)	47 (25.1)	63 (28.8)
**Member of RCGP**	381 (93.8)	115 (93.5)	266 (94.0)	138 (95.2)	243 (93.1)	175 (93.6)	206 (94.1)
**BMI category**							
Healthy weight (18–24.9 km/m^2^)	228 (56.2)	48 (39.0)	180 (63.6)	81 (55.9)	147 (56.3)	107 (57.2)	121 (55.3)
Overweight or obese (≥25 km/m^2^)	169 (41.6)	71 (57.7)	98 (34.6)	60 (41.4)	109 (41.8)	77 (41.2)	92 (42.0)
Missing	9 (2.2)	4 (3.3)	5 (1.8)	4 (2.8)	5 (1.9)	3 (1.6)	6 (2.7)
**Burnout**	144 (35.5)	50 (40.7)	94 (33.2)	45 (31.0)	99 (37.9)	60 (32.1)	84 (38.4)
**Psychological wellbeing category**							
Low	93 (22.9)	38 (30.9)	55 (19.4)	28 (19.3)	65 (24.9)	36 (19.3)	57 (26.0)
Medium	284 (70.0)	79 (64.2)	205 (72.4)	104 (71.7)	180 (69.0)	128 (68.4)	156 (71.2)
High	24 (5.9)	6 (4.9)	18 (6.4)	10 (6.9)	14 (5.4)	21 (11.2)	3 (1.4)
Missing	5 (1.2)	0 (0.0)	5 (1.8)	3 (2.1)	2 (0.8)	2 (1.1)	3 (1.4)

BMI = body mass index.

### Burnout, psychological wellbeing, and musculoskeletal health

More than one-third of GPs were classified as experiencing feelings of burnout (*n* = 144, 35.5%) (Table 1). Of those not experiencing burnout, most reported feeling under stress and not having as much energy as they once did (*n* = 216, 53.2%). Most GPs reported experiencing pain in their neck, shoulders, and back (*n* = 332, 81.8%), while half reported pain in their legs (*n* = 202, 49.8%) and almost one-third in their arms (*n* = 115, 28.3%). On average, the musculoskeletal pain score was the highest for the lower back, followed by the hip or thigh, upper back, and neck. When categorised, most GPs (*n* = 284, 70.0%) received a medium score on the psychological wellbeing scale and almost one-quarter were classified as experiencing low levels of psychological wellbeing (*n* = 93, 22.9%) (Table 1).

### Sitting time and physical activity

Almost two-thirds of GPs (*n* = 261, 64.3%) spent most of their sitting time at work in prolonged bouts (≥30 minutes) and took one or fewer breaks in sitting per hour (*n* = 219, 53.9%) (Table 1). Almost two-thirds of GPs (*n* = 262, 64.5%) reported meeting the current guidance for aerobic physical activity and over one-third reported doing muscle strengthening physical activity twice per week as advised in the guidelines (*n* = 140, 34.5%).^14,15^ Only 30.3% (*n* = 123) met both the aerobic and strength guidance amounts of participation in physical activity.

### Associations between sitting and physical activity and GPs’ health

GPs who met the combined aerobic and muscle-strengthening physical activity guidelines had a significantly lower burnout score compared with those who did not (*P* = 0.046). On average, GPs who had ≥2 breaks in sitting per hour had a significantly lower burnout score (*P* = 0.007) and higher psychological wellbeing score (*P* = 0.001) than those who had ≤1 breaks per hour. Spending ≥50% of sitting time in prolonged bouts (≥30 minutes) was also associated with a significantly higher prevalence of musculoskeletal complaints in the neck, shoulders, and back (*P* = 0.010) and in the legs (*P* = 0.021), compared with <50% of sitting time being spent in prolonged bouts (Table 2). Multiple linear regression modelling supported these associations, with the exception of the association between time spent sitting in prolonged bouts and the prevalence of musculoskeletal complaints in the neck, shoulders, and back (*P* = 0.310) (see Supplementary Table S1). Gender was the only significant predictor for neck, shoulder, and back pain (*P*<0.001), with more females (*n* = 249, 85.9%) reporting pain in this area than males (*n* = 81, 69.8%). No other demographic or work-related variables were significant predictors of any outcome.

**Table 2.** Differences in burnout, psychological wellbeing scores, and musculoskeletal complaints between physical activity and sitting status

		Physical activity status^a^			Prolonged sitting^a^			Breaks in sitting^a^		
**Category**		Meeting guidelines(*n* = 123)	Not meeting guidelines(*n* = 283)	*P* value	<50% of sitting(*n* = 145)	≥50% of sitting(*n* = 261)	*P* value	≥2 per hour(*n* = 187)	≤1 per hour(*n* = 219)	*P* value
**Burnout, mean (SD)^b^**		2.28 (0.79)	2.46 (0.87)	0.046	2.28 (0.81)	2.36 (0.82)	0.361	2.21 (0.82)	2.43 (0.80)	0.007
**Psychological wellbeing, mean (SD)^b^**		22.87 (3.32)	22.21 (3.09)	0.062	22.95 (3.11)	22.51 (3.33)	0.191	23.26 (3.74)	22.15 (2.68)	0.001
**MSK, *n* (%)^c^**	NSB pain	234 (57.6)	98 (24.1)		109 (26.8)	223 (54.9)		148 (36.5)	184 (45.3)	
	No NSB pain	49 (12.1)	25 (6.2)	0.470	36 (8.9)	38 (9.4)	0.010	39 (9.6)	35 (8.6)	0.205
	Arm pain	75 (18.5)	40 (9.9)		35 (8.6)	181 (44.6)		48 (11.8)	67 (16.5)	
	No arm pain	208 (51.2)	83 (20.4)	0.216	110 (27.1)	80 (19.7)	0.163	139 (34.2)	125 (30.8)	0.272
	Leg pain	146 (36.0)	56 (13.8)		61 (15.0)	141 (34.7)		86 (21.2)	116 (28.6)	
	No leg pain	137 (33.7)	67 (16.5)	0.262	84 (20.7)	120 (29.6)	0.021	101 (24.9)	103 (25.4)	0.161

^a^Musculoskeletal percentages are calculated from total sample responses: *n* = 406. ^b^Burnout and psychological wellbeing display means and standard deviations (SDs) following an independent *t*-test. ^c^MSK variables report frequencies and percentages following χ^2^ tests. MSK = musculoskeletal. NSB = neck, shoulder, and back.

### Time spent working each day or week

All GPs worked between 3 and 12 hours per day. The time spent working per day was not associated with burnout, psychological wellbeing, or musculoskeletal complaints. A large proportion of GPs did report long working hours, with the majority (*n* = 211, 81%) reporting working for >7.5 hours per day. A substantial number (*n* = 155, 38.2%) of GPs reported working for ≥9.6 hours per day, while 9.4% (*n* = 55) of GPs reported working for ≥11 hours per day.

Most GPs reported working as a GP for 3 or 4 days per week. The number of days worked per week was not associated with burnout, psychological wellbeing, or musculoskeletal complaints.

### Associations between time worked and days worked and GPs’ health

There was no difference in burnout, psychological wellbeing, or musculoskeletal complaints between groups of different working time or number of days worked (Table 3). Results were unchanged when assessed using multiple linear regression modelling that adjusted for demographic variables.

**Table 3.** Differences in burnout, psychological wellbeing scores, and musculoskeletal complaints between hours of work per day and number of days worked per week

		Hours worked per day^a^			Days worked per week^a^		
**Category**		≤9 hours(*n* = 197)	>9 hours(*n* = 188)	*P* value	≤3 days(*n* = 180)	>3 days(*n* = 226)	*P* value
**Burnout, mean (SD)^b^**		2.26 (0.85)	2.43 (0.79)	0.054	2.26 (0.79)	2.39 (0.84)	0.090
**Psychological wellbeing, mean (SD)^b^**		22.83 (3.12)	22.58 (3.23)	0.458	22.68 (3.55)	22.65 (3.02)	0.940
**MSK, *n* (%)^c^**	NSB pain	164 (42.6)	152 (39.5)		148 (36.5)	184 (45.3)	
	No NSB pain	33 (8.6)	36 (9.4)	0.540	32 (7.9)	42 (10.3)	0.834
	Arm pain	62 (16.1)	43 (11.2)		50 (12.3)	65 (16.0)	
	No arm pain	135 (35.1)	145 (37.7)	0.058	130 (32.0)	161 (39.7)	0.827
	Leg pain	94 (24.4)	93 (24.2)		97 (23.9)	105 (25.9)	
	No leg pain	103 (26.8)	95 (24.7)	0.731	83 (20.4)	121 (29.8)	0.137

^a^Percentages are calculated from: hours worked per day, *n* = 385; and days worked per week, *n* = 406. ^b^Burnout and psychological wellbeing display means and standard deviations (SDs) following an independent *t*-test. ^c^MSK variables report frequencies and percentages following χ^2^ tests. MSK = musculoskeletal. NSB = neck, shoulder, and back.

## Discussion

### Summary

In this study, a large proportion of GPs reported that they were experiencing burnout, with an alarmingly high number (81.8%) also experiencing neck, shoulder, and back pain. The majority of GPs reported that they spent most of their sitting time in prolonged bouts (64.3%) and did not meet the physical activity guidelines for health benefits (69.7%).^15^ GPs who had more regular breaks from sitting per hour reported significantly lower burnout and higher psychological wellbeing than those who had fewer breaks. Working within general practice may put GPs at risk of physical and psychological morbidity, yet meeting physical activity guidelines and reducing prolonged periods of sitting more regularly may help to improve these outcomes.

### Strengths and limitations

Strengths include the novel nature of this study and the large sample of GPs who were recruited from across all four nations of the UK. The sample was largely representative, with a similar demographic profile as the wider GP population across the UK.^16^ The questionnaire captured GPs’ thoughts during a period of increase in COVID-19 transmission and rapid changes to government-imposed restrictions across the UK. This study is not without limitations. The response rate was modest although a broad range of GPs working in different parts of the UK were recruited and participants were well balanced across gender, ethnic group, and job role. Nevertheless, it should be acknowledged that the response rate to this study is susceptible to some potential bias whereby GPs who were feeling less burnt out and more psychologically healthy may have been more willing to respond to the survey. This would suggest that the findings may represent a better case scenario than what is experienced by GPs more broadly in the UK. Self-reported physical activity can be over-reported and sitting time under-reported, compared with when these behaviours are measured using a device,^17^ although the self-reported questionnaires used here have been validated against device-based measures. The cross-sectional nature of these data prevent causality being concluded.

### Comparison with existing literature

A recent systematic review and meta-analysis examined feelings of burnout among GPs and concluded there was a moderate-to-high prevalence of burnout worldwide.^18^ The present study supports this finding, with a substantial number of GPs reporting feelings of burnout. The previous review suggested that the working environment for GPs may influence the prevalence of burnout. However, the present study points towards behavioural factors (physical activity and sedentary behaviour) as important drivers of burnout, rather than work-related factors (for example, hours worked per day). It is possible that general practice could be altered to promote physical activity in the workplace, by encouraging regular breaks in sitting and/or encouraging a healthier work–life balance. This may be one strategy to help reduce feelings of burnout among GPs.

As expected, sitting for prolonged periods of time was associated with a higher prevalence of musculoskeletal complaints. GPs meeting current public health guidelines for physical activity per week were significantly less likely to experience burnout relative to their less physically active counterparts, although the difference was small. This study, and the learning from previous pandemics, has highlighted that the health of GPs can be adversely affected during such times.^19–21^ In general, but particularly during a pandemic, it is critical that healthcare workers remain physically and mentally able to work, to ensure there is sufficient workforce available to treat and attend to the medical needs of the public. Strategies to support GPs’ health are needed and the promotion of health-protecting behaviours, such as participation in physical activity and reducing sitting time, are two ways in which this might be achieved.

The study findings are consistent with strong evidence that has consistently demonstrated that physical activity is important for maintaining good health.^14,15^ More specifically, participation in physical activity is known to enhance mental health outcomes such as stress, anxiety, and depression, as well as being effective for the prevention and treatment of musculoskeletal complaints and disorders.^22,23^ Current evidence has also suggested sedentary behaviour is associated with increased risk of depression and anxiety.^24,25^

This study did not show any differences in burnout, psychological wellbeing, and musculoskeletal complaints between those who work less or more than 9 hours per day, or between those who work for less or more than 3 days per week. However, 81.0% of GPs reported working for >7.5 hours per day, with 38.2% working >9.6 hours per day. Of note, hours and/or days worked was not related to musculoskeletal complaints, although gender was significant, with females experiencing more of these types of complaints. No associations were found for burnout or psychological wellbeing with working hours or working days. These results further highlight the importance of increasing physical activity and/or reducing sitting time for GPs’ health and wellbeing, as these associations appear to be independent of GPs’ characteristics or working environment.

### Implications for practice

A high proportion of GPs reported experiencing burnout and musculoskeletal complaints, but these health concerns were less evident in GPs who spent less time in prolonged sitting, took more breaks in sitting, and who were more physically active. Workplace health initiatives in primary care should consider how working practices might negatively impact the health and wellbeing of GPs during pandemics. This will be particularly important if remote consultations are to continue to be a large part of how GPs consult with their patients, and the demands on primary healthcare services continue to increase.  

## Data

The dataset relied on in this article is available from the corresponding author on reasonable request.

## References

[1] British Medical Association (2023). An NHS under pressure. https://www.bma.org.uk/advice-and-support/nhs-delivery-and-workforce/pressures/an-nhs-under-pressure.

[2] Hippisley-Cox J, Vinogradova Y (2009). Trends in consultation rates in general practice 1995 to 2008: analysis of the QResearch database. https://files.digital.nhs.uk/publicationimport/pub01xxx/pub01077/tren-cons-rate-gene-prac-95-09-95-08-rep.pdf.

[3] Hobbs FDR, Bankhead C, Mukhtar T (2016). Clinical workload in UK primary care: a retrospective analysis of 100 million consultations in England, 2007–14. Lancet.

[4] Owen K, Hopkins T, Shortland T, Dale J (2019). GP retention in the UK: a worsening crisis. findings from a cross-sectional survey. BMJ Open.

[5] Haynes L (2020). Increasing numbers of GPs suffering burnout or depression during COVID-19 outbreak. https://www.gponline.com/increasing-numbers-gps-suffering-burnout-depression-during-covid-19-outbreak/article/1683827.

[6] Jefferson L, Golder S, Heathcote C (2022). GP wellbeing during the COVID-19 pandemic: a systematic review. Br J Gen Pract.

[7] Jefferson L, Heathcote C, Bloor K (2023). General practitioner well-being during the COVID-19 pandemic: a qualitative interview study. BMJ Open.

[8] Biddle GJH, Thomas N, Edwardson CL (2022). The views of GPs about using sit-stand desks: an observational study. BJGP Open.

[9] Cooke GPE, Doust JA, Steele MC (2013). A survey of resilience, burnout, and tolerance of uncertainty in Australian general practice registrars. BMC Med Educ.

[10] Ng Fat L, Scholes S, Boniface S, Mindell J (2017). Evaluating and establishing national norms for mental wellbeing using the short Warwick-Edinburgh Mental Well-being Scale (SWEMWBS): findings from the Health Survey for England. Qual Life Res.

[11] Kuorinka I, Jonsson B, Kilbom A (1987). Standardised Nordic questionnaires for the analysis of musculoskeletal symptoms. Appl Ergon.

[12] Quiles NN, McCullough AK, Piao L (2019). Validity and reliability of the exercise vital sign questionnaire in an ethnically diverse group: a pilot study. J Prim Care Community Health.

[13] Chau JY, Van Der Ploeg HP, Dunn S (2012). Validity of the occupational sitting and physical activity questionnaire. Med Sci Sports Exerc.

[14] Department of Health and Social Care, Llwodraeth Cymru Welsh Government, Department of Health Northern Ireland, Scottish Government (2019). UK Chief Medical Officers’ physical activity guidelines. https://assets.publishing.service.gov.uk/media/5d839543ed915d52428dc134/uk-chief-medical-officers-physical-activity-guidelines.pdf.

[15] Bull FC, Al-Ansari SS, Biddle S (2020). World Health Organization 2020 guidelines on physical activity and sedentary behaviour. Br J Sports Med.

[16] General Medical Council (2018). What our data tells us about general practitioners working for the NHS in England and Scotland. https://www.gmc-uk.org/-/media/documents/what-our-data-tells-us-about-gps_pdf-74830685.pdf.

[17] Prince SA, Adamo KB, Hamel ME (2008). A comparison of direct versus self-report measures for assessing physical activity in adults: a systematic review. Int J Behav Nutr Phys Act.

[18] Karuna C, Palmer V, Scott A (2022). Prevalence of burnout among GPs: a systematic review and meta-analysis. Br J Gen Pract.

[19] Wong WCW, Lee A, Tsang KK, Wong SYS (2004). How did general practitioners protect themselves, their family, and staff during the SARS epidemic in Hong Kong. J Epidemiol Community Health.

[20] Verma S, Mythily S, Chan YH (2004). Post-SARS psychological morbidity and stigma among general practitioners and traditional Chinese medicine practitioners in Singapore. Ann Acad Med Singap.

[21] Austin Z, Martin JC, Gregory PAM (2007). Pharmacy practice in times of civil crisis: the experience of SARS and the blackout in Ontario, Canada. Res Social Adm Pharm.

[22] Kandola A, Ashdown-Franks G, Hendrikse J (2019). Physical activity and depression: towards understanding the antidepressant mechanisms of physical activity. Neurosci Biobehav Rev.

[23] Garber CE, Blissmer B, Deschenes MR (2011). American College of Sports Medicine position stand. Quantity and quality of exercise for developing and maintaining cardiorespiratory, musculoskeletal, and neuromotor fitness in apparently healthy adults: guidance for prescribing exercise. Med Sci Sports Exerc.

[24] Teychenne M, Ball K, Salmon J (2010). Sedentary behavior and depression among adults: a review. Int J Behav Med.

[25] Teychenne M, Costigan SA, Parker K (2015). The association between sedentary behaviour and risk of anxiety: a systematic review. BMC Public Health.

